# Disorder of sex development: a rare case of a boy with an XY karyotype and Magnetic Resonance Imaging findings of hermaphroditism

**DOI:** 10.5935/1518-0557.20220072

**Published:** 2023

**Authors:** Rosaria Meucci, Mario Bengala, Guglielmo Manenti, Francesca Montesanto, Chiara Palombi, Flavia Rufi, Carolina Goffredo, Eleonora Lombardo, Maria Lina Serio, Roberto Floris

**Affiliations:** 1 UOC Diagnostica per immagini, Policlinico Tor Vergata, University Rome “Tor Vergata”, Rome, Italy; 2 Genetica Medica, Policlinico Tor Vergata, University Rome “Tor Vergata”, Rome, Italy

**Keywords:** disorder of sex development, intersex disorder, true hermaphroditism, magnetic resonance imaging

## Abstract

Disorders of sexual differentiation are rare congenital conditions in which the chromosomal, anatomic or gonadal sex development is atypical. In some of these patients, chromosomal sex is inconsistent with phenotypic sex; in other cases, the phenotype is not classifiable as either male or female, resulting in a condition known as ambiguous genitalia. These are very complex cases in which diagnostic certainty is not always possible. A multidisciplinary team including geneticists, pediatricians, radiologists is certainly needed to approach these patients. We present the case of an 18-year-old boy with an XY karyotype, ambiguous genitalia, uterus and blind-ending vaginal pouch. The patient had not been previously diagnosed with a disorder of sex development. The patient underwent a panel of genetic analyses and diagnostic imaging investigations. Magnetic resonance imaging was decisive for the identification of the internal genital organs, especially the uterus. At the end of investigations, the patient was diagnosed with 46,XY disorder of sex development. Our purpose is to underline the role of imaging in the diagnosis and management of congenital disorders of sex differentiation.

## INTRODUCTION

Disorders of sex development (DSD) are congenital conditions in which the chromosomal, anatomic or gonadal sex development is atypical.

### Classification and Prevalence

DSD can be classified as disorders of chromosomal, gonadal, or phenotypic sex origin.

Today, DSD is divided into three major categories based on patient karyotype: sex chromosome DSD; 46,XY DSD; and 46,XX DSD. The etiology of DSD is multifaceted and and may include genetic and environmental factors (Kohva *et al*., 2018). In particular, 46,XY DSD patients are genetically male and constitute a more heterogeneous group, representing a spectrum from normal appearing females to males with hypospadias and infertility. These patients may have underdevelopment of the genital tubercle (hypospadias and/or micropenis) with or without undescended gonads, with or without feminine remnants (Müllerian structures). Within this group are patients with:

- Disorders of gonadal (testicular) development (true hermaphroditism)
- Disorders of androgen synthesis or action:
  - Defects in androgen biosynthesis (e.g.: 17-hydroxysteroisd dehydrogenase deficiency, 5a reductase deficiency)
  - Defects in androgen action (e.g.: complete androgen insensitivity syndrome)
  - LH receptor defects (e.g.: Leydig cell hypoplasia, aplasia)
  - Disorders of Anti-Müllerian hormone (AMH) and AMH receptor (Persistent Müllerian Duct Syndrome)
  - Other (e.g.: severe hypospadias, cloacal exstrophy, exposure to androgens during fetal life) (Calvo *et al*., 2016)

On the basis of gonadal histologic features, these disorders were originally divided into four broad groups: female pseudohermaphroditism, male pseudohermaphroditism, true hermaphroditism, and gonadal dysgenesis. When the external genitalia do not have the typical anatomic appearance of normal male or female genitalia, the condition is known as ambiguous genitalia (Chavhan *et al*., 2008). The incidence of DSD varies among ethnic groups with the highest incidence in southern African populations (Witchel, 2018).

### Main features and diagnosis

In general, DSD is defined as a condition in which chromosomal sex is inconsistent with phenotypic sex or in which the phenotype is not classifiable as either male or female. Abnormalities may result in abnormal differentiation of the gonads, internal genital ducts, or external genitalia. These abnormalities result in predictable clinical syndromes (Mansour *et al*., 2012). While many of these defects of sex differentiation are evident at birth, others are not identified until puberty, at which time the patient may manifest aberrant external maturation or remain sexually infantile and obviously infertile (Hughes *et al*., 2006).

Most causes of DSD are recognized in the neonatal period. Later presentations in older children and young adults, as in our case, are rare (Hughes *et al*., 2006).

Diagnostic algorithms do exist, but with the spectrum of findings and diagnoses, no single evaluation protocol can be recommended in all circumstances.

First-line testing includes: karyotyping with X- and Y-specific probe detection (even when prenatal karyotype is available); imaging (abdomin-pelvic ultrasound US and Magnetic Resonance Imaging) (Hughes *et al*., 2006). Imaging plays a key role in the evaluation of patients with DSD. The choice of imaging method varies according to age and clinical presentation. The first-line imaging method is ultrasound, followed in some cases by genitography and MRI (Caprio *et al*., 2019). In many cases, ultrasonography does not allow the adequate identification of internal genitalia. Thus, in recent years clinicians have employed MRI as the gold standard imaging exam to diagnose patients with the condition. It does not use ionizing radiation and is more sensitive in the definition of spatial relations and characterization of tissues. It also allows the evaluation of all associated anatomic anomalies (renal, skeletal, bone marrow) (Caprio *et al*., 2019).

### Risk of cancer

Malignant change is reported in 2.6% of gonads (Walker *et al.,* 2000).

### Treatment

Managing patients with DSD and their families is enormously challenging, owing to the diagnostic and ethical challenges present in many cases (especially in individuals with 46,XY DSD) and the difficulty offering a precise prognosis for individual patients (Hiort *et al*., 2014). The use of a multidisciplinary team can play a key role in targeting treatment to improve the health and well-being of DSD patients and their families.

The surgical approach to early DSD has traditionally involved early reconstructive surgery of the external genitalia to make their appearance consonant with the chosen gender assignment. Recent changes in practice dictate that only surgeons with expertise on the complex procedures of genital reconstruction and knowledge of longer-term outcomes should be involved in early management of DSD (Hughes, 2007). The surgeon is responsible for providing an outline of the sequence of surgical procedures and the various consequences that may materialize in later childhood and early adulthood. Others issues which also need addressing later include further surgical procedures around the time of puberty, the risk of gonadal tumors, and options for gonadectomy, sexual function and the potential for fertility.

Hormonal treatment primarily involves pubertal induction of hypogonadism, hormone replacement therapy (HRT) at various ages and, in some instances, pubertal suppression. Pubertal induction is usually performed at ages 10-12 in girls and 11-13 in boys, depending on maturity, desire, and informed consent of patients and parents. Options for hormonal treatment for patients with DSD are limited by practical considerations, such as pharmacokinetic properties and effectiveness of steroid hormone preparations, and availability (Lee *et al*., 2016). HRT and surgery should be offered only after a full psychological evaluation at the appropriate age to fully informed patients. There is no consensus regarding the indications, timing, and extent of surgery for individuals with DSD.

There is evidence that the definition of the native sex and acceptance of sexuality differs significantly between societies. Therefore, when discussing sex-related issues with the family, one should not overlook social, cultural, ethnic, and religious aspects of the family or society. In fact, cultural markers that give an individual the notion that they are male or female are fundamental, but changeable according to prevailing cultural patterns. For example, Chinese tradition stipulates that boys carry on the family lineage. Therefore, most conservative families prefer male to female children after thorough evaluation and worry about the catastrophic effect of gender reassignment on the whole family (Mao *et al*., 2017).

In our culture, parents of DSD newborns usually want their children to undergo genital surgery as soon as possible after sexual assignment, as surgery helps them to confirm the assigned sex. When the diagnosis of DSD is made during childhood and adolescence or in older individuals, patient participation in decision-making becomes more obvious. This is the case of children raised as girls in whom testicles are found at the time of surgery for bilateral inguinal hernias; or of adolescents with primary amenorrhea or absent development of breasts or virilization; or teenagers raised as boys showing signs of breast development. Importantly, data regarding the outcomes of individuals with DSD not treated during childhood are more limited.

## CLINICAL CASE

An 18-year-old boy born in Somalia was taken to the emergency unit with hyperpyrexia, vomiting, and dysuria. A language barrier prevented the obtention of his full medical history. The patient was phenotypically male and physical examination revealed a man with hypospadias and a difficult-to-identify urethral meatus, testicular hypotrophy with the left testicle on site and an unpalpable right testicle, gynecomastia and retained nipples, gynoid fat, and no facial or limb dysmorphism. He had never been diagnosed with DSD. The patient was sent for diagnostic investigation for suspicion of DSD and true hermaphroditism in particular.

**Figure 1:**
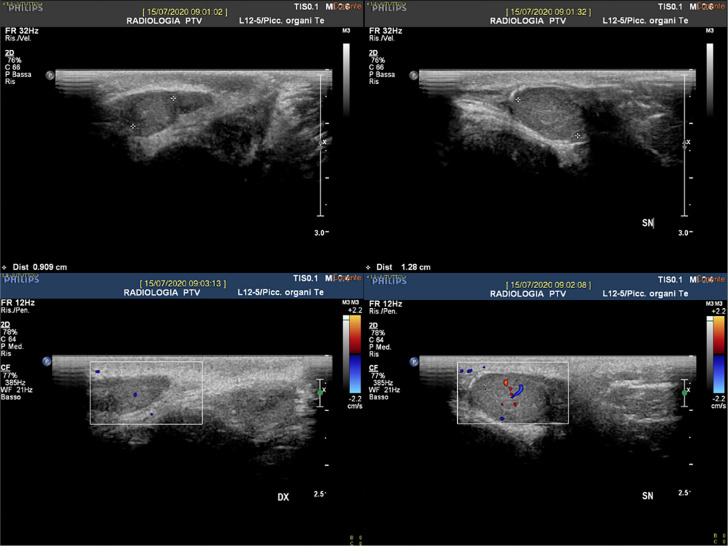
Lower abdomen and testicular US: testicles on site and smaller than normal (left 15x8mm and right 10x6mm), asymmetric vascularity (reduced to the right).

Blood tests revealed altered hormone levels with LH 31.01 mlU/ml (v.n. mans 0.57-12.97) and FSH 53.66 Mui/ml (v.n. mans 0.95-11.95). Estradiol and testosterone levels were normal (25.00 pg/Ml and 350.88 pg/dL, respectively) (Tanner stage II). Lower abdominal and testicular ultrasound images did not show a prostate or uterus and ovaries; the testicles were on site and were smaller than average (left 15x8mm and right 10x6mm) with asymmetric vascularity, reduced to the right. Epididymis could not be evaluated (Figure 1). A pelvic MRI using a 1.5-T magnet was performed (Philips Intera Achieva, Best, the Netherlands). The patient was imaged in the supine position using a pelvic phased-array coil. Standard pelvic MRI protocols included T1-weighted, T2-weighted, T2-STIR weighted and DWI images.

**Figure 2:**
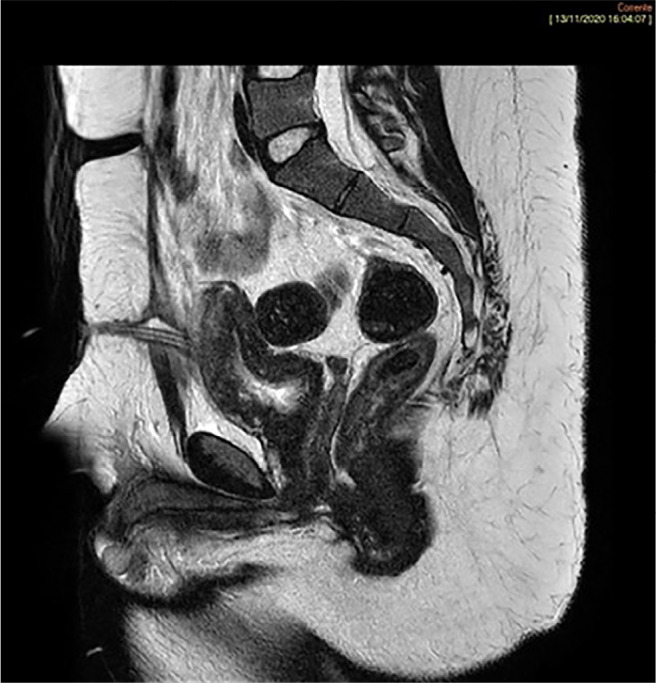
T2-weighted sagittal image: Uterine remnants between rectum and bladder characterized by low to medium signal intensity in T2-w with indistinct zonal anatomy. Blind-ending vaginal pouch.

**Figure 3:**
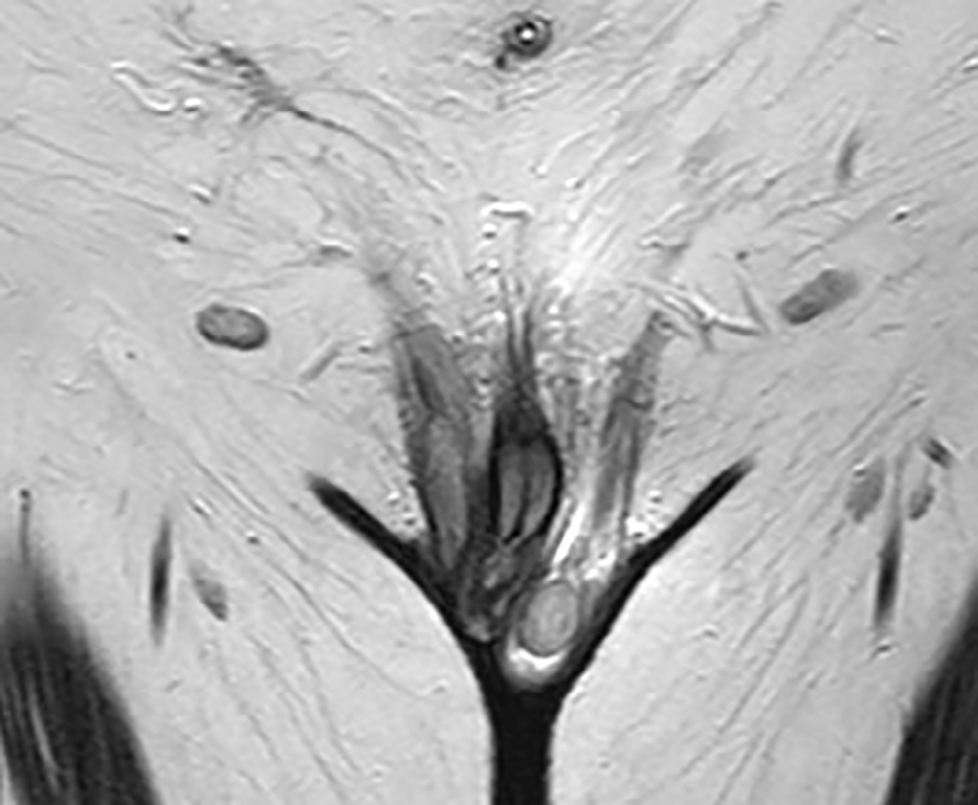
T2-weighted coronal image: hypertrophic clitoris with cavernosa corpora (penis-like clitoris); and left testicle.

**Figure 4:**
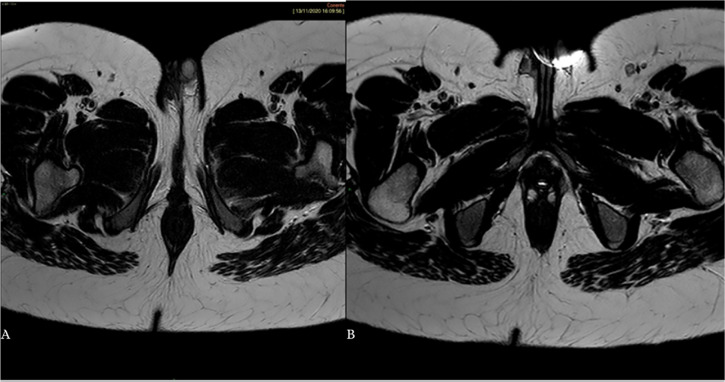
T2-weighted axial image: left (A) and right (B) testicles. Bilateral labioscrotal fold with hyperintense, asymmetrical and small testicles (left testis larger than right).

The imaging reports described the following: a vagina ending in a blind pouch in the perineum between the urethra and rectum; uterine remnants between the rectum and the bladder (Figure 2); starting from the fundus, two symmetrical linear formations were documented with hypointense images in T1-w and T2-w, referring to immature tubal formations or broad ligament remnants; a hypertrophic clitoris, consisting of corpora cavernosa (penis-like clitoris) (Figure 3); the corpus spongiosum cannot be seen, consistent with a possible case of aplasia; Bilateral labioscrotal folds, in which two testicular formations are appreciated, measuring 15x10mm on the left and 7x5mm on the right (Figure 4).

**Figure 5:**
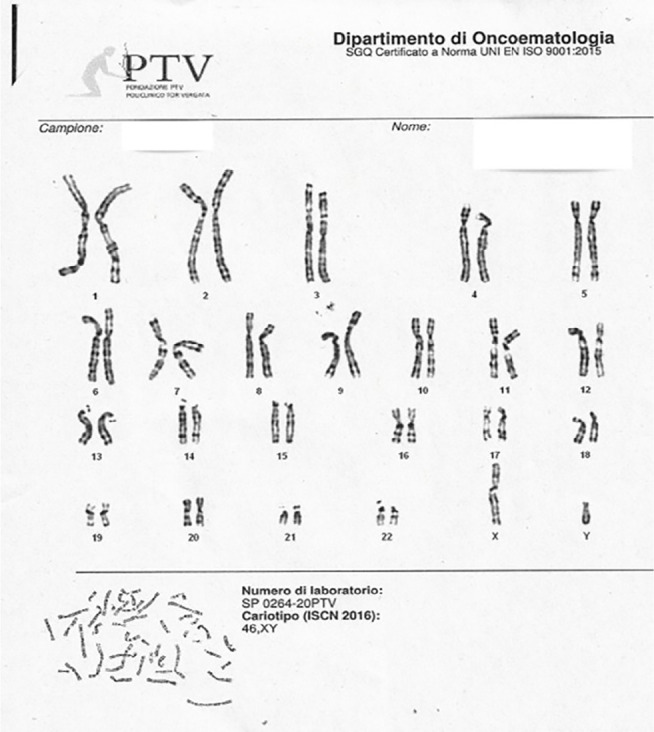
karyotype analysis: karyotype 46,XY.

Genetic counseling is required. After informing the patient, the decision was made to perform karyotype analysis. Peripheral blood was collected and analyzed with short-term lymphocyte culture (48-72 hours). The result was a normal male karyotype 46,XY (Figure 5). The following additional investigations were performed:

Analysis by Multiplex Ligation-dependent Probe Amplification (MLPA) to search for gene duplications/deletions *SOX9, NR0B1(DAX1), WNT4, NR5A1 (SF1)*;

Next-Generation Sequencing (NGS) of the coding exons and related junctions of the 27 genes involved in sexual differentiation.

No anomaly was found.

## DISCUSSION

Pelvic ultrasonography (US) is the most frequently used imaging method in the initial assessment of DSD in patients of all ages.

Magnetic resonance imaging (MRI) and US are considered equally sensitive in the evaluation of intrapelvic structures, although MRI is more sensitive than US in the evaluation of the gonads (Chavhan *et al*., 2008). The presence of gonads cannot be ruled out in cases where they are not viewed in imaging. Laparoscopic examination of the pelvic structures might be required in these cases. In some cases, biopsy of intra-abdominal gonads is required in the management of intersex disorders (Calvo *et al*., 2016).

MRI is useful in the evaluation of ambiguous genitalia, with identification of the uterus in 93%, the vagina in 95%, the penis in 100%, the testes in 88%, and one ovary in 74% of the cases (Gambino *et al*., 1992).

Gonadal composition may comprise ovary and testis, ovary and ovotestis, bilateral ovotestis, ovotestis and testis gonads (Chavhan *et al*., 2008). Walker *et al*. (2000) demonstrated that the gonadal combination of ovotestis and ovary is the most common, followed by bilateral ovotestis. Ovaries are more common on the left side (76.5% left, 23.5% right), while gonads containing testicular tissue occur more often on the right (60.3% right, 39.7% left). The likelihood of gonadal descent was dependent on the amount of testicular tissue present (more testicular tissue increasing the chance of descent).

Testicular ultrasound revealed our patient had smaller-than-average testicles. The adult testis diameter ranges between 30-50 mm *vs*. 10-15 mm in our patient. We could not evaluate the epididymis; in normal adults, the epididymis is clearly visible both in US and MRI. MRI confirmed the presence of bilateral labioscrotal folds with a left testis larger than the right, but no ovaries were observed.

Our patient’s karyotype was 46,XY. Differential diagnosis included 46,XY DSDs. Literature reports indicate that 46,XX karyotype occurs most frequently (71%), followed by mosaicism (Zou *et al*., 2022) (usually 46,XX/46,XY, 20%), while 46,XY occurred less often (7%) (Walker *et al*., 2000). Furthermore, in our case both Müllerian and wolffian structures were present and the external genitalia was atypical; the absence of ovaries on MRI does not rule out a diagnosis of true hermaphroditism (Chavhan *et al*., 2008).

As reported in the literature, in our case ectopic gonads had intermediate signal intensity on T1-weighted images and intermediate signal intensity on T2-weighted images. Streak gonads are difficult to detect and can be seen as low intensity stripes on T2-weighted images of the gonads (Chavhan *et al*., 2008). Our patient had uterine remnants, in which a trilaminar zonal anatomy was not identifiable, with low to medium signal intensity in T2-w with an indistinct zonal anatomy typical of a premenarchal or postmenopausal uterus (Langer *et al*., 2012).

The external genitalia presented as a penis-like clitoris; for Chavhan *et al*. (2008), clitoral hypertrophy can be differentiated from a penis through MRI on the basis of absent or poorly developed supporting penile structures, such as the bulbospongiosus muscle and posteriorly located transverse perineal muscles; moreover, we could not see a prostate in our patient in either US or MRI.

We have not found any disorders in androgen synthesis or action. No pathogenetic variants of genes implicated in DSD were found. Thus, the most probable diagnosis, although not certain, is true hermaphroditism. True hermaphroditism is a DSD defined as the presence of ovarian tissue and testicular tissue in the same individual.

Definitive diagnosis requires an exploratory laparoscopy, an invasive procedure not always accepted by patients. It is not uncommon for these patients to remain without a definitive diagnosis; in fact, some studies have shown that only 50% of 46,XY children with DSD will receive a definitive diagnosis (Calvo *et al*., 2016).

## CONCLUSION

The management of DSDs is complex and requires a multidisciplinary medical approach. Medical decisions for patients with DSD still lack evidence-based principles, and an early and correct diagnosis of DSD carries important clinical implications for patient upbringing, the reduction of potential risk of malignancy, and the decision on the correct timing for a gonadectomy.

It is important that a child with ambiguous genitalia be evaluate by a multidisciplinary team using a coordinated approach to arrive at a timely diagnosis, so that proper gender assignment can be made early in life. Imaging plays an important role in demonstrating the anatomy and potential effects on other organs, in particular when a pathognomonic genetic alteration is not identified, as in our case.

US is the first line exam for the evaluation of DSD. Our case underlines that MRI is needed in most cases for a correct diagnosis of gonadal morphological features, especially when a pathognomonic genetic alteration is not identified. MRI does not involve exposure to ionizing radiation, allows multiplanar image reconstruction, and provides excellent soft tissue contrast resolution.

Radiologists play an essential role in diagnosis and follow-up, considering the high risk of tumors developing in these patients. US every six months or MRI every year is recommended to patients with dysgenetic gonads.
